# Early DAS response after DMARD-start increases probability of achieving sustained DMARD-free remission in rheumatoid arthritis

**DOI:** 10.1186/s13075-020-02368-9

**Published:** 2020-11-23

**Authors:** M. Verstappen, E. Niemantsverdriet, X. M. E. Matthijssen, S. le Cessie, A. H. M. van der Helm-van Mil

**Affiliations:** 1 Department of Rheumatology, Leiden University Medical Center, P.O. Box 9600, 2300 RC Leiden, the Netherlands; 2 Department of Clinical Epidemiology, Leiden University Medical Center, Leiden, the Netherlands; 3 Department of Medical Statistics and Bioinformatics, Leiden University Medical Center, Leiden, the Netherlands; 4 Department of Rheumatology, Erasmus Medical Center, Rotterdam, the Netherlands

**Keywords:** Rheumatoid arthritis, DMARD-free remission, Drug-free remission, Disease activity scores, Anti-citrullinated protein antibodies

## Abstract

**Background:**

Sustained DMARD-free remission (SDFR) is increasingly achievable. The pathogenesis underlying SDFR development is unknown and patient characteristics at diagnosis poorly explain whether SDFR will be achieved. To increase the understanding, we studied the course of disease activity scores (DAS) over time in relation to SDFR development. Subsequently, we explored whether DAS course could be helpful identifying RA patients likely to achieve SDFR.

**Methods:**

772 consecutive RA patients, promptly treated with csDMARDs (mostly methotrexate and treat-to-target treatment adjustments), were studied for SDFR development (absence of synovitis, persisting minimally 12 months after DMARD stop). The course of disease activity scores (DAS) was compared between RA patients with and without SDFR development within 7 years, using linear mixed models, stratified for ACPA. The relation between 4-month DAS and the probability of SDFR development was studied with logistic regression. Cumulative incidence of SDFR within DAS categories (< 1.6, 1.6–2.4, 2.4–3.6, ≥ 3.6) at 4 months was visualized using Kaplan-Meier curves.

**Results:**

In ACPA-negative RA patients, those achieving SDFR showed a remarkably stronger DAS decline within the first 4 months, compared to RA patients without SDFR; − 1.73 units (95%CI, 1.28–2.18) versus − 1.07 units (95%CI, 0.90–1.23) (*p* < 0.001). In APCA-positive RA patients, such an effect was not observed, yet SDFR prevalence in this group was low. In ACPA-negative RA, DAS decline in the first 4 months and absolute DAS levels at 4 months (DAS_4 months_) were equally predictive for SDFR development. Incidence of SDFR in ACPA-negative RA patients was high (70.2%) when DAS_4 months_ was < 1.6, whilst SDFR was rare (7.1%) when DAS_4 months_ was ≥ 3.6.

**Conclusions:**

In ACPA-negative RA, an early response to treatment, i.e., a strong DAS decline within the first 4 months, is associated with a higher probability of SDFR development. DAS values at 4 months could be useful for later decisions to stop DMARDs.

## Key messages

- Despite RA’s chronic character, sustained DMARD-free remission is increasingly achievable in both in ACPA-negative and ACPA-positive RA. The biological pathways underlying SDFR-development are poorly understood. Additionally, identified baseline characteristics poorly explain which RA patients are able to achieve SDFR over time.
- This is the first study scoping beyond baseline characteristics to understand SDFR, by exploring the course of disease activity scores (DAS) over time in relation to achieving SDFR.
- This study showed that early response to treatment, i.e., significant decrease in DAS within the first 4 months after diagnosis, is associated with achieving SDFR in ACPA-negative RA. DAS levels in the first 4 months were predictive for SDFR development, and incidence of SDFR was high when DAS < 1.6 at 4 months, whilst SDFR was rare when DAS was ≥ 3.6 at 4 months.
- Our findings suggest that early reduction of inflammation can influence chronification in ACPA-negative RA, i.e., the development of SDFR, and that the “window of opportunity” might expand to the early phase after initiation of DMARD treatment.
- Evaluation of early response to treatment might be helpful for clinicians in later decisions to stop DMARDs in ACPA-negative RA.

## Background

Rheumatoid arthritis (RA) is regarded as a chronic disease, requiring life-long treatment [1]. Nonetheless, tapering and sustained cessation of DMARDS, termed sustained DMARD-free remission (SDFR), is increasingly achievable in ACPA-negative and ACPA-positive RA [2, 3]. Achieving SDFR has been shown to associate with symptom resolution and normalization of functional ability [2]. Also, it limits prolonged exposure to side-effects and unnecessary healthcare costs [4]. Currently, SDFR is the best proxy for disease resolution (or “cure”) in RA [5].

So far, little is known about the biological pathways underlying the development of this favorable long-term outcome [6]. Several patient characteristics at time of diagnosis have been studied [2, 7–10], but these poorly explained SDFR development. Only the presence of auto-antibodies was repeatedly found to be unfavorable for SDFR development [2, 7, 8, 11–13]. This relates to the existence of two subsets of RA patients, in which those with auto-antibodies have a lower capability of achieving SDFR [3, 12]. Different mechanisms underlying SDFR development might be involved in ACPA-positive and ACPA-negative RA patients. It has been suggested that SDFR development in ACPA-negative RA patients solely reflects spontaneous resolution of inflammation in patients misclassified as RA (e.g., reactive arthritis or osteoarthritis). Yet, since SDFR is also achievable in ACPA-positive RA, it cannot solely depend on misclassification of disease. Furthermore, the finding that SDFR has become more frequent with improved treatment strategies, also in ACPA-negative RA [2], indicates that SDFR is a real disease outcome in RA patients that otherwise would have had a chronic disease.

Measures of inflammation (joint counts, acute phase reactants, MRI-detected joint inflammation) at the time of diagnosis appeared non-informative in distinguishing which RA patients are likely to achieve SDFR [2, 7–10, 13, 14]. Potentially, differences in biological pathways leading towards disease resolution, i.e., SDFR, are not detectable at baseline, but might unfold later on. Then, not disease characteristics at diagnosis, but during the disease course might relate to SDFR development. It could be hypothesized that timely suppression of inflammation with DMARD treatment can influence chronicity of disease, i.e., benefit SDFR development. However, there is currently no longitudinal data available which could provide insight in the course of inflammation over time and SDFR development. It has been reported that timely response to treatment is beneficial for other outcomes in RA, like sustained remission and radiographic progression [15–19]. Potentially, timely response to DMARD treatment can also benefit SDFR development in RA patients.

Difficulty in identifying RA patients that are highly likely to achieve SDFR makes clinicians ambiguous in their decisions to discontinue DMARD treatment. EULAR guidelines suggest to consider tapering, but do not provide further practical guidance for treatment discontinuation [4, 20]. Because discontinuation of DMARD treatment in clinical practice is currently based on trial and error, it is essential to attain further knowledge on SDFR development in both ACPA-positive and ACPA-negative RA. Effective prediction of successful achievement of SDFR will safeguard patients from flare, which has indisputable impact on health status, psychological health, and social life [21, 22].

With the ultimate aim to increase the understanding of SDFR development in RA, and acknowledging that measures of inflammation at the time of diagnosis are of little importance for this outcome, we explored the relation between disease activity over time with achieving SDFR. Subsequently, we explored whether information on the course of disease activity may be relevant for distinguishing which RA patients are more likely to achieve SDFR.

## Patients and methods

### Patient selection

Patients for this study were obtained from the Leiden Early Arthritis Clinic (EAC), which has been previously described [23]. In short, the Leiden EAC is an inception cohort, including all patients presenting with recent onset arthritis with a symptom duration ≤ 2 years. For this study, all consecutive RA patients fulfilling the 1987 and/or 2010 criteria [24, 25] and promptly treated with conventional DMARDs, were evaluated. RA was evaluated after 1 year of follow-up and stringently defined by a clinical diagnosis by an experienced rheumatologist plus fulfillment of 1987/2010 criteria. Patients diagnosed with conditions other than RA (e.g., reactive arthritis, psoriatic arthritis, inflammatory osteoarthritis) during follow-up were excluded. Although the cohort started in 1993, prompt DMARD treatment, and subsequent tapering when remission was achieved, became common since 1999; therefore, RA patients that were consecutively included from 1999 onwards were used. Patients who participated in clinical trials (and therefore not routinely treated) were excluded.

### Follow-up

Research visits took place at baseline, after 4 months and annually afterwards. During these visits, joint counts were performed, laboratory measurements done (serum samples tested for CRP level, ESR, IgG ACPA (ELISA CCP (anti-CCP2), Phadia, Nieuwegein, the Netherlands), and IgM rheumatoid factor (RF; in-house ELISA [26])) and questionnaires filled out. Four-component formula was used to calculate DAS [27]. Follow-up duration differed among RA patients (median 7.0 years, IQR, 4.1–11.6 years), which is inherent to the design of an observational cohort. All available follow-up data was studied, but SDFR achievement was assessed after 7 years.

### Treatment

In brief, all RA patients were promptly treated with csDMARDs after diagnosis, in which methotrexate was the first choice. DAS-steered treatment adjustments became common since 2005. When treatment with initial conventional DMARD (csDMARD) failed, another csDMARD was initiated or added. A biological DMARD (bDMARD) was allowed when RA patients failed ≥ 2 csDMARDs. If clinical remission (defined as DAS < 2.4) was sustained, and clinical synovitis was absent, treatment could be tapered and eventually discontinued. Decisions on cessation of DMARDs were taken in shared-decision making between rheumatologists and patients.

### Outcome

Sustained DMARD-free remission (SDFR) was defined as the absence of clinical synovitis (swollen joints at physical examination) after discontinuation of DMARD treatment (including systemic and intra-articular corticosteroids) that persisted for the entire follow-up thereafter and this follow-up should be ≥ 1 year. This stringent definition was chosen to ensure sustainability. Medical files were studied on occurrence of SDFR until May 2017.

Using all available data up to 7 years of follow-up, two outcome groups were discerned; RA patients achieving SDFR within 7 years, and those who did not. RA patients achieving SDFR after 7 years were regarded as non-SDFR. Additionally, to be stringent in sustainability of the outcome, RA patients experiencing a flare (defined as recurrence of clinical synovitis) after SDFR development were also included in the non-SDFR group.

### Statistical analysis

Baseline characteristics were compared using *t* test, Mann-Whitney *U* test and Fisher’s exact test as appropriate.

The course of DAS over 7 years was compared between the SDFR group and non-SDFR group, using linear mixed models. Splines were used to model the non-linear relation between DAS and time [28], with knots at baseline, 4 m, 12 m, and for every single year after, based on the structured research visits. Analyses were repeated for the individual DAS components (SJC, TJC, ESR, VAS). Additionally, analyses were stratified for ACPA status.

Subsequently, to evaluate whether DAS scores were predictive for SDFR development within 7 years, the associations between DAS decline between 0 and 4 months (∆DAS_0–4m_) and absolute 4-month DAS levels (DAS_4 months_) with SDFR development were explored using logistic regression models, with SDFR development as dependent variable. Baseline characteristics were added to construct multivariable models. Analyses were stratified for ACPA. Thereafter, to incorporate differences in a follow-up, and time to SDFR development, Kaplan-Meier curves were constructed and used to determine cumulative incidence of SDFR within 7 years for different DAS categories at 4 months. DAS_4 months_ levels were categorized as follows; < 1.6, 1.60–2.39, 2.4–3.59, and ≥ 3.6.

Two sensitivity analyses were done. First, patients achieving SDFR after 7 years were also included in the SDFR group, i.e., grouping all RA patients ever achieving SDFR together, and LMM analysis were repeated. Similarly, patients with a flare after 7 years, but achieving SDFR< 7 years, were included in the SDFR group to compare results.

The second sub-analyses concerned the evaluation of missing data for the analysis on the predictive ability of the DAS_4 months._ So far, only complete data was used (*n* = 365). In 177 RA patients, the DAS_4 months_ was incomplete due to missing DAS components, that were unrecorded during research visits, and in 230 RA patients DAS_4 months_ was missing completely due to unattended visits. Baseline characteristics of patients with missing DAS_4 months_ did not remarkably differ from those with complete DAS_4 months_ (*n* = 365) (supplementary Table S1). Missing DAS scores or DAS components were imputed using chained equations (MICE) [29]. Point estimates and confidence intervals were pooled according to Rubin’s rules across 30 imputations.

STATA (V12.0), SPSS (V.25.0), and R (V3.5.2) were used. *P* values< 0.05 were considered statistically significant.

## Results

### Study population

Between January 1999 and December 2014, 1109 RA patients were consecutively included in the EAC and promptly treated with csDMARDs. 337 patients were excluded due to concomitant participation in a clinical trial. Consequently, 772 RA patients were included in this study (supplementary Figure S2), which all had a clinical diagnosis of RA and fulfilled the 1987 and/or 2010 criteria. Compared to the study population, patients that were excluded because of trial participation had higher disease activity and higher rate of ACPA positivity at baseline (supplementary Table S3).

Of the 722 included RA patients, 45% was ACPA-positive, of the 400 ACPA-negative patients, 24.3% were positive for rheumatic factor (RF).

Within the total study population, 149 RA patients achieved SDFR within 7 years after diagnosis (SDFR group), after median 3.2 years (IQR, 2.0–4.6 years). Twenty-four patients achieved SDFR after 7 years (median 9.6 years (IQR, 7.4–10.5)) and were included in the non-SDFR group, together with the 588 patients who never achieved SDFR during follow-up. Additionally, 11 patients flared after SDFR development; these flares occurred relatively late after achieving SDFR (median 2.6 years (IQR, 2.3–5.5)). These patients were included in the non-SDFR group to strictly define the SDFR group. Baseline characteristics are presented in Table 1.

**Table 1:** Baseline characteristics of the study population

	Total study population (*n* = 772)	non-SDFR group (*n* = 623)	SDFR group (*n* = 149)
Age (years), mean (SD)	58.0 (15.4)	56.6 (15.2)	64.0 (14.9)
Females, *n* (%)	528 (68.4)	435 (70.7)	88 (59.1)
ACPA positivity^+^ *n* (%)	348 (45.1)	333 (54.1)	15 (10.1)
Symptom duration at diagnosis^+^ *(≤ 12* vs *> 12 weeks), n* (%)	257 (33.5)	207 (33.2)	50 (33.6)
DAS at baseline^+^, med (IQR)	3.10 (2.52–3.72)	3.10 (2.51–3.69)	3.10 (2.55–3.91)
SJC at baseline^+^, (0–44), med (IQR)	6 (3–11)	6 (3–11)	8 (4–13)
TJC at baseline^+^, (0–53), med (IQR)	6 (4–9)	6 (4–10)	6 (4–9)
ESR^+^ (mm/h), med (IQR)	29 (14–45)	29 (14–45)	29 (14–47)
VAS^+^ (0–100 mm), med (IQR)	40 (20–60)	41 (20–60)	38 (20–60)

*Legend*: Baseline characteristics of the patients with RA in the total study population and subgroups according to achievement of sustained DMARD-free remission (SDFR) during 7 years of follow-up. ^+^ACPA status was missing in 24 patients. Symptom duration was missing in 50 patients. DAS at baseline was missing in 124 patients. DAS components SJC, TJC, ESR, and VAS were missing in 29, 124, 8, and 101 patients

*DAS* disease activity score based on swollen joint count (44 joints), tender joint count (53 joints), ESR, and pain; *SJC* swollen joint count; *TJC* tender joint count, *ESR* estimated sedimentation rate; *VAS* visual analog scale, *ACPA* anti-citrullinated protein antibody

### Disease activity scores over time in relation to SDFR

**Fig. 1:**
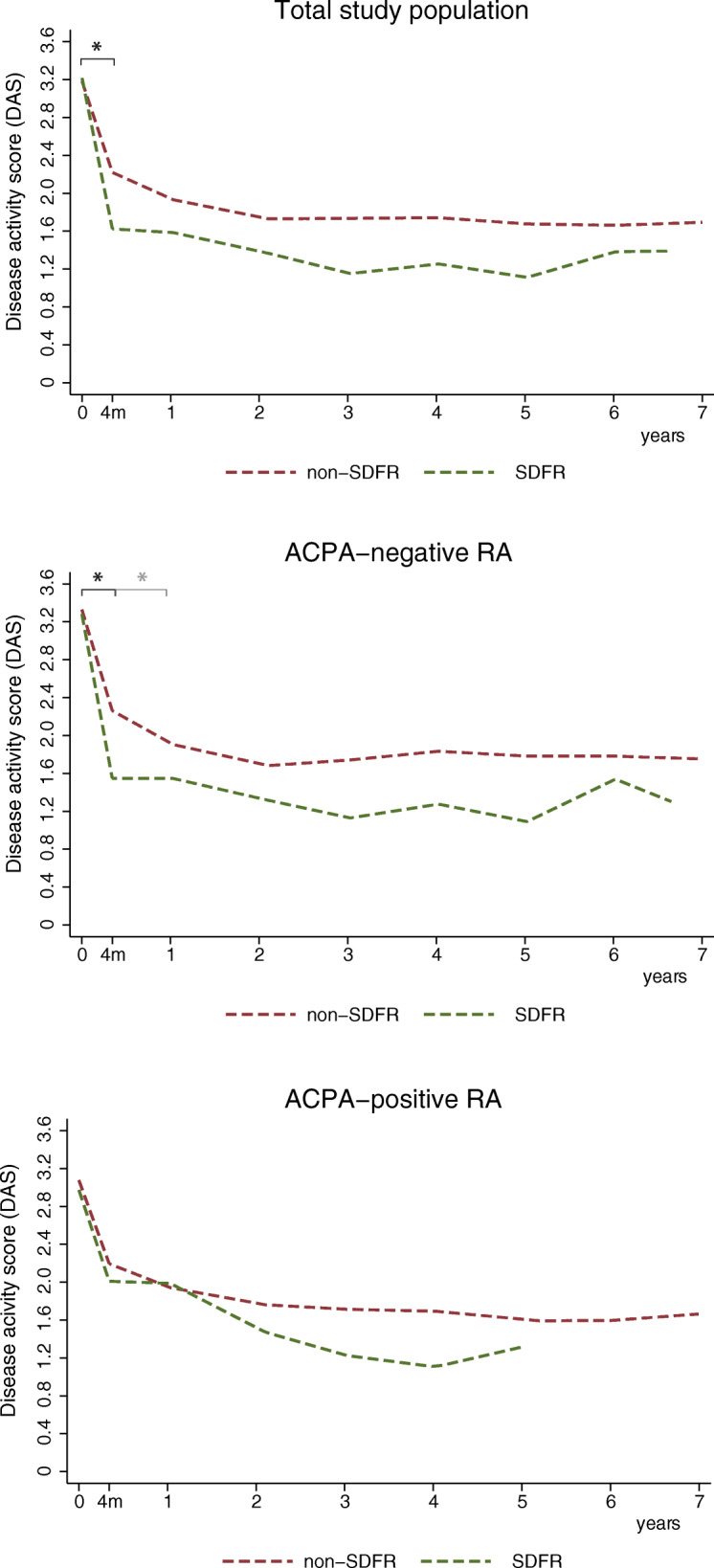
Course of DAS over time for the SDFR and non-SDFR group in the total RA population studied, and for ACPA-negative RA and ACPA-positive RA separately. *Legend:* Course of DAS over time of patients achieving sustained DMARD-free remission (SDFR) within 7 years of follow-up (*n* = 149), and those not (*n* = 623). In ACPA-positive patients, the line of the SDFR group was restricted to 5 years of follow-up because of insufficient data thereafter. Statistically significant differences in course of DAS over time, between the SDFR group and non-SDFR group, were indicated by with *. ACPA anti-citrullinated protein antibody, DAS disease activity scores, RA rheumatoid arthritis, SDFR sustained DMARD-free remission

Baseline DAS was not different between the SDFR and non-SDFR groups (Fig. 1, supplementary Table S4). However, the SDFR group showed a stronger decline in DAS within the first 4 months; − 1.59 units (95%CI; 1.24–1.95) versus − 0.96 units (95%CI, 0.85–1.07) in the non-SDFR-group (*p* < 0.001) (Fig. 1). No significant differences in DAS course after 4 months were seen between both groups (supplementary Table S4).

### DAS components over time in relation to SDFR

**Fig. 2:**
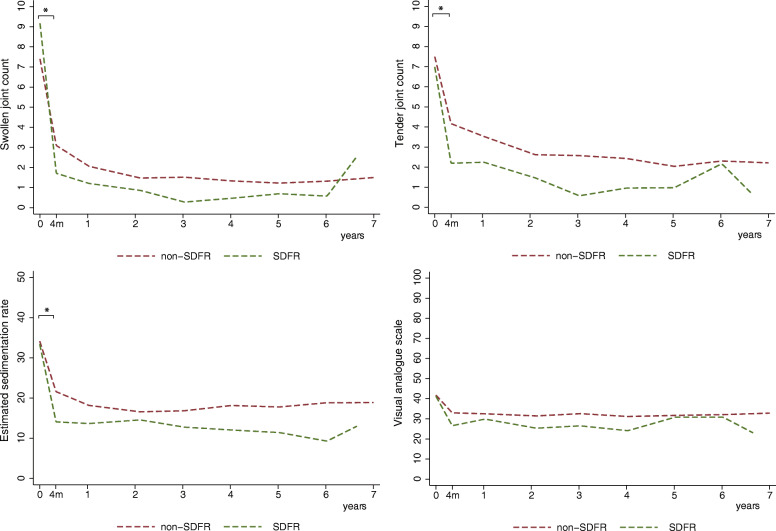
Course of DAS components over time for the SDFR- and non-SDFR group in all RA patients. *Legend:* Course of DAS components over time of patients achieving sustained DMARD-free remission (SDFR) within 7 years of follow-up, and those not. Statistically significant differences in course of DAS over time, between the SDFR group and non-SDFR group, were indicated by with *. DAS disease activity scores, RA rheumatoid arthritis, SDFR sustained DMARD-free remission

Analyses were repeated for the individual DAS components. RA patients achieving SDFR had a statistically significant stronger decline in SJC, TJC, and ESR within the first 4 months, compared to RA patients who did not achieve SDFR (*p* < 0.001) (Fig. 2, supplementary Table S5). Course of VAS did not significantly differ between both groups.

### Stratification for ACPA

In ACPA-negative RA patients, 31.8% (127/400) achieved SDFR within 7 years, after a median 3.2 years of follow-up (IQR, 1.7–4.5 years). LMM analysis in ACPA-negative RA showed that patients achieving SDFR had a stronger decline in DAS within the first 4 months; − 1.73 units (95%CI; -2.18, -1.28) versus − 1.07 units (95%CI; − 1.23, − 0.90) (*p* < 0.001) (Fig. 1, supplementary Table S4). In the interval from 4 to 12 months, the non-SDFR group showed a slightly stronger decline, suggestive of a delayed DAS response compared to the SDFR group.

ACPA-positive RA patients achieved SDFR in 4.3% (15/348), median 3.3 years after diagnosis (IQR, 1.9–4.8 years). In ACPA-positive RA, both groups showed a decline in DAS within the first 4 months (Fig. 2), but this decline was not stronger in the SDFR group; − 0.88 units (95%CI, − 1.03, − 0.73) versus − 0.96 units (95%CI; − 1.76, − 0.15). Later time intervals did not show large differences (supplementary Table S4). Notably, SDFR prevalence in the ACPA-positive RA group was low, which might have limited the power to detect differences in ACPA-positive patients achieving SDFR.

### Early DAS response as a predictor for achieving SDFR

Subsequently, we explored whether DAS levels at 4 months can be used to predict SDFR development within 7 years. In uni- and multivariable logistic regression analysis in ACPA-negative RA (*n* = 171), a stronger decrease in DAS between baseline and 4 months (ΔDAS_0–4m_), i.e., a more negative ΔDAS_0–4m_, was associated with an increased chance of SDFR development. Inversely, a smaller decline in DAS decreased the chance of SDFR: OR0.58 (95%CI; 0.42–0.80) for one point less decline in DAS (Table 2, supplementary Table S6).

**Table 2:** Association of DAS_0–4m_ and DAS_4m_ with SDFR development within 7 years in ACPA-negative RA

	Multivariable logistics regression models					
	OR (95%CI)	*p* value	OR (95%CI)	*p* value	OR (95%CI)	*p* value
Age (years)	1.01 (0.99–1.04)	0.37	1.02 (0.99–1.04)	0.38	1.02 (0.99–1.05)	0.13
Gender (male)	1.65 (0.80–3.39)	0.18	1.40 (0.66–2.97)	0.31	1.25 (0.61–2.57)	0.55
Symptom duration at diagnosis *(≤ 12 weeks* vs. *> 12 weeks)*	0.93 (0.45–1.88)	0.82	0.87 (0.42–1.79)	0.70	0.97 (0.49–1.93)	0.92
Baseline DAS	–		1.44 (1.00–2.08)	0.05	–	
DAS_4 months_	**–**		**0.44 (0.29–0.68)**	**< 0.01**	**0.51 (0.35–0.75)**	**< 0.01**
ΔDAS_0–4m_	**0.58 (0.42–0.80)**	**< 0.01**	**–**		**–**	

*Legend:* Multivariable logistic regression models in ACPA-negative RA (*n* = 184) with SDFR (yes/no) as dependent outcome variable. Multivariable analysis showed DAS levels at 4 months (DAS_4 months_) were as predictive of SDFR as decrease in ΔDAS_0–4m_, with and without incorporation of baseline DAS in the model

*DAS* disease activity score, *BL* baseline, *CI* confidence interval

Since DAS at one time point is more practical for use in clinical practice than a DAS change over time, absolute DAS levels at 4 months (DAS_4 months_) were studied, and included in the model instead of ΔDAS_0–4m_. In ACPA-negative RA patients, absolute DAS_4 months_ was predictive for SDFR development, OR0.44 (95%CI, 0.29–0.68), in a model that included baseline DAS (this analysis still mimicked ΔDAS_0–4 m_), and also in a model without baseline DAS, OR0.51 (95%CI, 0.35–0.75) (Table 2, supplementary Table S6). Thus, ΔDAS_0–4m_ and DAS_4 months_ were equally predictive for SDFR development.

**Fig. 3:**
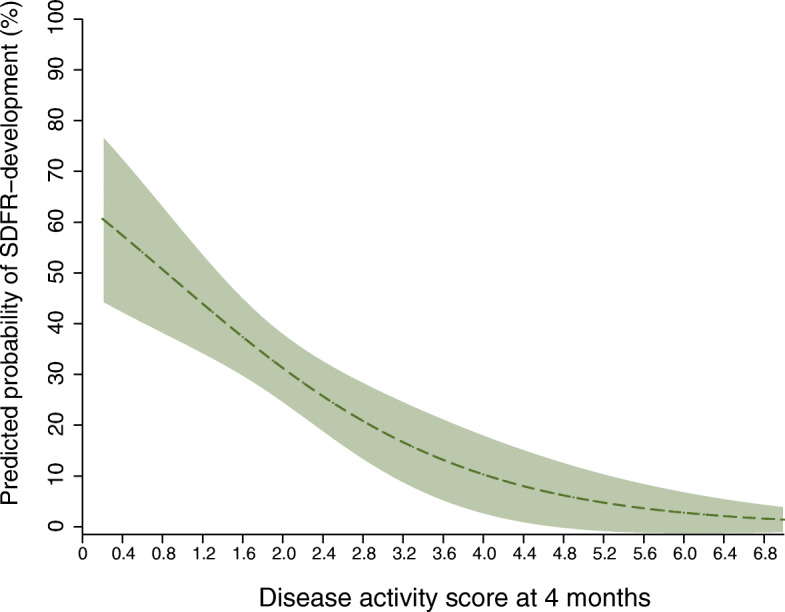
Predicted probability of SDFR development in ACPA negative RA in relation to DAS at 4 months. *Legend:* Predicted probability of achieving SDFR during follow-up in ACPA-negative RA, based on multivariate logistic model including DAS at 4 months, age, gender, and symptom duration (Table 2). For this graph, age, gender, and symptom duration were set at the mean value of each covariable. An inverse relation is seen between DAS at 4 months and the predicted probability of achieving SDFR (within 7 years). ACPA anti-citrullinated protein antibody, DAS disease activity score, SDFR sustained DMARD-free remission

Figure 3 depicts the inverse relation between DAS_4 months_ and the predicted probabilities of achieving SDFR, based on the multivariable logistic model without baseline DAS.

**Fig. 4:**
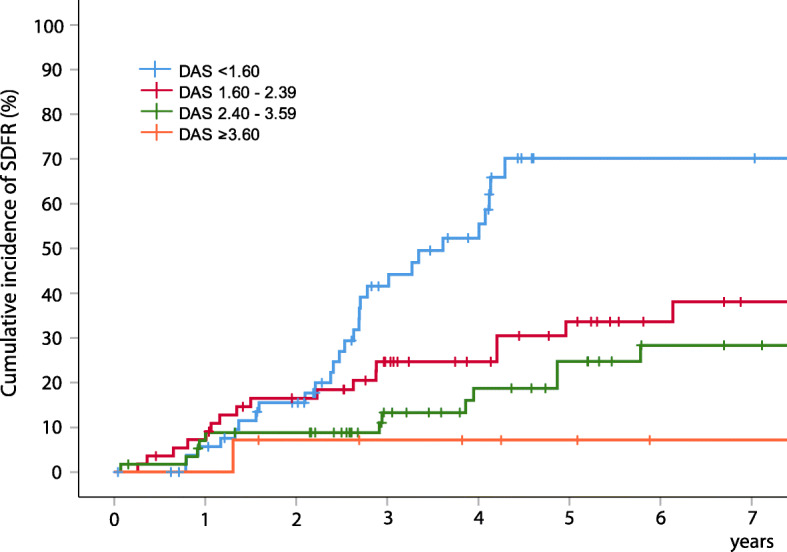
Kaplan-Meier curves for SDFR development within 7 years of follow-up based on disease activity scores at 4 months in ACPA-negative RA. *Legend:* Time-to-event was defined as time from 4 months visit until SDFR development (yes/no), i.e., the absence of clinical arthritis for minimally 12 months after DMARD stop. ACPA anti-citrullinated protein antibody, DAS disease activity score, SDFR sustained DMARD-free remission

Subsequently, Kaplan-Meier curves were constructed, categorizing patients in 4 groups based on DAS_4 months_ (< 1.6, 1.60–2.39, 2.4–3.59, ≥ 3.6) (Fig. 4). The cumulative incidence among ACPA-negative RA patients with DAS_4 months_ < 1.6 was high (70.2%), whereas SDFR was rare (7.1%) among patient with DAS_4 months_ ≥ 3.6.

In ACPA-positive RA (*n* = 169), incidence of SDFR development was low, and neither ΔDAS_0–4m_ nor DAS_4 months_ was associated with SDFR development (supplementary Table S6). The ACPA-positive RA patients achieving SDFR (*n* = 15) had a median DAS_4 months_ of 2.22 units (IQR, 2.19–2.37) with a median ΔDAS_0–4m_ of − 0.60 units (IQR, − 0.96, − 0.50). This did not significantly differ from the ACPA-positive RA patients not achieving SDFR; DAS_4 months_ 2.22 units (IQR, 1.52–2.84) and ΔDAS_0–4m_ − 0.80 units (IQR, − 1.50, − 0.05).

### Sensitivity analyses

Including patients achieving SDFR after 7 years (*n* = 24) in the SDFR group, instead of the non-SDFR group, and repeating the LMM yielded similar results on the course of DAS between the two groups. Including patients that flared after 7 years, but achieved SDFR< 7 years, in the SDFR group also yielded similar results (supplementary Tables S7 and S8).

Imputing the missing DAS data and repeating logistic regression analyses and Kaplan-Meier analysis yielded similar results (supplementary Table S9 and Figure S10).

## Discussion

SDFR, the sustained absence of clinical synovitis after DMARD stop, reflects resolution of disease chronicity and is currently the best possible long-term outcome for RA. The chance to achieve this outcome is not well predicted by DAS at diagnosis but, as we demonstrated here, is strongly associated with a rapid decline in DAS in the first months after diagnosis and treatment start. The notion that an early response to treatment is important for this long-term outcome suggests that timely reduction of inflammation might confine chronification in RA. Moreover, the comprehension that DAS levels at 4 months are predictive for the ability to achieve SDFR over time in ACPA-negative RA patients can be important for clinicians, as it can contribute to the selection of RA patients eligible for DMARD discontinuation. Especially 4-month DAS of < 1.6 or ≥ 3.6 may be considered as most relevant, as these values were associated with high and low incidence rates of SDFR, respectively.

The importance of early clinical response for outcome in RA has been emphasized in previous research [15–19]. A relation between early clinical response and achieving low disease activity or remission after 6 or 12 months [15, 16, 18], thus short-term outcomes, has been reported. Following, early remission appeared beneficial for sustainability of remission during DMARD-treatment as reported previously [19]. However, limited data is available on the association with long-term outcomes in RA, such as SDFR. The IMPROVED study [11] reported early remission to be associated with a higher occurrence of SDFR in UA-/RA-population. However, patients achieving DAS remission at 4 months were treated differently from those who did not achieve remission at 4 months. This makes comparison of SDFR, among those with and without early remission, conditional. In our study, we expand on previous findings with more detailed analyses on time courses and on possible clinical utility. Also, in our study, all patients were treated conform current guidelines, comparable to most clinical practice.

The finding that DAS change within the first months is related to achieving SDFR, suggests that biological pathways relevant for SDFR development evolve in the earliest phase after diagnosis and treatment initiation in ACPA-negative RA. Timely suppression of inflammation might confine chronification. According to the “window of opportunity”, disease processes mature over time and early intervention is therefore more effective. The present results may suggest that the timeline of the window of opportunity does not comprise the period before diagnosis but also extends in the early period directly after diagnosis and treatment initiation in ACPA-negative RA. To unravel such mechanisms, further studies could focus on the early phase after start of DMARD treatment. Interestingly, several cytokines were recently observed to be increased in patients that achieved SDFR over time [30], such observations now merits further longitudinal studies in samples repeatedly taken early after treatment start.

Our findings were inconclusive in ACPA-positive RA since the low SDFR prevalence in these patients limited the power for our analysis. However, no clear tendency towards an effect was observed. Whether a higher SDFR prevalence in our ACPA-positive group would have resulted in a significant effect remains warranted. In our study, tapering was non-protocolized (and based on shared decision making), which might have underestimated the SDFR prevalence. This underestimation may be more present in ACPA-positive RA if either patients or rheumatologists more frequently did not want to stop DMARDs because of the believe that ACPA-positive RA is a more severe disease and cessation would be unsuccessful [31]. Nevertheless, previous research, applying protocolized tapering, also showed SDFR prevalence in ACPA-positive RA was low [32]. Following, ACPA positivity seems to increase flare rate after achieving DMARD-free remission [33, 34]. Altogether this suggests that pathways leading towards SDFR might be different for ACPA-positive and ACPA-negative RA. Potentially, the hypothesized “extension” of the window of opportunity does not hold for ACPA-positive RA patients since these have a longer pre-arthritis phase [35] and, possibly, disease processes have fully matured at the time of diagnosis and initiation of treatment. Yet, this remains subject to future research.

It has been suggested that SDFR development in ACPA-negative patients solely reflects spontaneous resolution of inflammation in patients misclassified as RA. To rule out this possibility, we only analyzed patients who had a clinical RA diagnosis after 1 year of follow-up, and patients diagnosed with conditions other than RA during follow-up were excluded. Additionally, all included patients fulfilled the 1987 and/or 2010 criteria, in which more than half (58%) of the ACPA-negative patients even fulfilled both criteria. Thus, all included patients had both a clinical diagnosis of RA and fulfilled classification criteria, which are meant for research as presented here. Additionally, of the 127 ACPA-negative RA patients who achieved SDFR, 16% was RF-positive and 22% erosive at baseline, supporting that the ACPA-negative RA SDFR group did not solely contain patients with least severe RA.

Also within RA, it has been implied that SDFR as treatment outcome is non-existent, but reflects spontaneous remission [36], in which “early response” could be regarded part of this natural course. Our study was observational in nature, and did not include a placebo arm, and unfortunately is unable to address this question. However, it is known that DAS levels most prominently decline in the first months after treatment initiation [28]. The DAS course in untreated RA patients is relatively unexplored.

Currently, treatment and tapering strategies are formally similar for ACPA-positive and ACPA-negative RA patients [4, 20], and discontinuation of treatment is based on trial and error. Our findings might aid to construct more substantiated discontinuation strategies, stratified for ACPA status. We showed that ACPA-negative RA patients who rapidly achieve DAS remission < 1.6 have a considerably chance of future successful tapering and cessation of DMARD therapy. The optimal moment for evaluation of an early treatment response remains to be determined. In our cohort, a protocolized visit took place after 4 months. A DAS decrease within an earlier period (e.g., first 3 months after diagnosis or even earlier) might also be predictive. Further research on timelines remains warranted.

A limitation of this study was the presence of missing DAS information at 4 months. This was partially due to missing DAS components unrecorded during research visits; we considered this as “missing completely at random” since missingness is based on logistical issues. DAS_4 months_ was also missing due to unattended 4 months of visits; this could be related to (unmeasured) patient or disease characteristics. Fortunately, baseline features of patients with and without missing data did not markedly differ and sensitivity analysis in which missing data was imputed showed similar results. Another limitation is that the choice to categorize patients in the SDFR group if the outcome was achieved < 7 years was relatively arbitrary and another follow-up time might also have been useful for categorization.

Strengths of our study are the sample size and the long-term follow-up that allowed to use a a strict definition of SDFR. The fact that (although ≥ 1 year of follow-up without recurrence of synovitis was required) the actual follow-up without DMARDs was much longer, 5.0 years (IQR, 2.7–9.2), shows the sustainability of this outcome. Patients that were followed for less than 7 years and did not achieve SDFR were categorized in the non-SDFR group; this harbors the risk of misclassification, as SDFR could have been achieved if 7 years of follow-up was completed. This may also have underestimated the SDFR prevalence in both the ACPA-negative and ACPA-positive RA patients and might have resulted in an underestimation of the observed effect between the SDFR and non-SDFR groups.

## Conclusion

In conclusion, an early decrease in clinical disease activity in ACPA-negative RA patients seems to confine chronicity of RA by increasing the chance of SDFR. This suggests that the window of opportunity expands early after diagnosis. Further pathogenetic studies in these phases are needed to unravel the mechanisms involved in resolution of disease persistence.

## Supplementary Information

**Additional file 1: Table S1.** Baseline characteristics of patients with a complete DAS at 4 months and patients with missing/incomplete DAS at 4 months. **Figure S2**. Flowchart diagram of the selection of study participants. **Table S3.** Baseline characteristics of excluded patients due to trial participation. **Table S4.** Results linear mixed model analysis of DAS over time. **Table S5.** Results linear mixed model analysis of DAS-components over time. **Table S6.** Logistic regression models (uni- and multivariable) for SDFR-development within 7 years. **Table S7.** Sensitivity analysis of all patients achieving SDFR during complete follow-up. **Table S8.** Sensitivity analysis of patients achieving SDFR within 7 years, but flaring after 7 years. **Table S9.** Logistic regression models with imputed data. **Figure S10.** Kaplan-Meier curves with imputed data (ACPA negative patients only).

## Data Availability

All data relevant to the study are included in the article or uploaded as supplementary information. Additional data are available upon reasonable request.
