# Supplementary material for: Early DAS response after DMARD-start increases probability of achieving sustained DMARD-free remission in rheumatoid arthritis
Source: Arthritis Res Ther. 2020 Nov 23;22:276. doi: 10.1186/s13075-020-02368-9 (PMC7684730; doi:10.1186/s13075-020-02368-9)
Supplement: Supplementary file 1 — Additional file 1: Table S1. Baseline characteristics of patients with a complete DAS at 4 months and patients with missing/incomplete DAS at 4 months. Figure S2. Flowchart diagram of the selection of study participants. Table S3. Baseline characteristics of excluded patients due to trial participation. Table S4. Results linear mixed model analysis of DAS over time. Table S5. Results linear mixed model analysis of DAS-components over time. Table S6. Logistic regression models (uni- and multivariable) for SDFR-development within 7 years. Table S7. Sensitivity analysis of all patients achieving SDFR during complete follow-up. Table S8. Sensitivity analysis of patients achieving SDFR within 7 years, but flaring after 7 years. Table S9. Logistic regression models with imputed data. Figure S10. Kaplan-Meier curves with imputed data (ACPA negative patients only). [file 13075_2020_2368_MOESM1_ESM.docx]

**SUPPLEMENTARY MATERIAL**

S1. Baseline characteristics of patients with a complete DAS at 4 months and patients with missing/incomplete DAS at 4 months

S2. Flowchart diagram of the selection of study participants

S3. Baseline characteristics of excluded patients due to trial participation

S4. Results linear mixed model analysis of DAS over time

S5. Results linear mixed model analysis of DAS-components over time

S6. Logistic regression models (uni- and multivariable) for SDFR-development within 7 years

S7. Sensitivity analysis of all patients achieving SDFR during complete follow-up

S8. Sensitivity analysis of patients achieving SDFR within 7-years, but flaring after 7-years

S9. Logistic regression models with imputed data

S10. Kaplan-Meier curves with imputed data (ACPA negative patients only)

**Supplementary table S1: Baseline characteristics of patients with a complete DAS at 4 months and patients with missing or incomplete DAS at 4 months**

|  | **Total study population**  (n = 772) | **DAS_4months_ complete**  (n = 365) | **DAS_4months_ missing or incomplete**  (n = 407) |
| --- | --- | --- | --- |
| Age(years), mean (SD) | 58.0 (15.4) | 58.2 (15.0) | 57.8 (15.8) |
| Females, n(%) | 528 (68.4) | 243 (66.6) | 285 (70.0) |
| ACPA positivity, n (%) | 348 (45.1) | 169 (46.3) | 179 (44.0) |
| Symptom duration at diagnosis  *(≤12 vs >12 weeks),* n (%) | 257 (33.5) | 142 (38.9)* | 115 (28.3)* |
| DAS at baseline, med (IQR) | 3.10 (2.52-3.72) | 3.12 (2.52-3.78) | 3.06 (2.51-3.68) |
| SJC at baseline(0-44), med (IQR) | 6 (3-11) | 6 (3-11) | 6 (3-11) |
| TJC at baseline(0-53), med (IQR) | 6 (4-9) | 6 (4-10) | 6 (4-9) |
| ESR(mm/h), med (IQR) | 29 (14-45) | 29 (14-43) | 29 (11-48) |
| Pain(VAS 0 -100 mm), med (IQR) | 40 (20-60) | 43 (20-60) | 49 (20-62) |

*Legend:* Baseline characteristics of patients with a complete DAS_4m_  (n=365) and those with incomplete or missing DAS_4months_ (n=407). Baseline characteristics were compared between patients with complete DAS at 4 months and patients with missing or incomplete DAS at 4 months. *p<0.05

*DAS: disease activity score based on swollen joint count (44 joints), tender joint count (68-joints), ESR and pain. SJC: swollen joint count, TJC: tender joint count, ESR: Estimated Sedimentation Rate, VAS: Visual Analogue Scale, ACPA: anti-citrullinated protein antibody*

*med: median, n: number*

**Supplementary figure S2: Flowchart diagram of the selection of study participants**


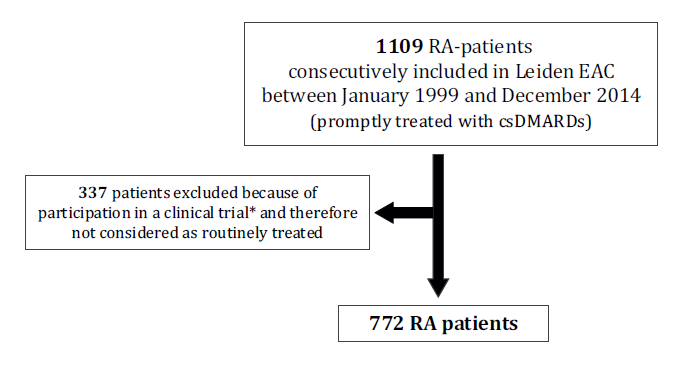


*Legend:* Flowchart of selection of study population.

* Among which: BeSt study, IMPROVED study and U-Act-Early study

*DMARD: Disease-modifying antirheumatic drugs, EAC: Early Arthritis Clinic, RA: Rheumatoid arthritis*

**Supplementary table S3: Baseline characteristics of excluded patients due to trial participation**

|  | **Total study population**  (n = 772) | **Exclusion due to trial participation**  (n=337) |
| --- | --- | --- |
| Age (years), mean (SD) | 58.0 (15.4) | 54.2 (14.1)* |
| Females, n (%) | 528 (68.4) | 218 (64.7) |
| ACPA positivity, (n. %) | 339 (44.5) | 173 (51.3)* |
| Symptom duration (weeks), med (IQR) | 18 (9-36) | 18 (10-36) |
| DAS at baseline, med (IQR) | 3.10 (2.52-3.72) | 3.24 (2.70-4.05)* |
| SJC at baseline(0-44), med (IQR) | 6 (3-11) | 7 (3-12) |
| TJC at baseline(0-53), med (IQR) | 6 (4-9) | 7 (5-11)* |
| ESR (mm/h), med (IQR) | 29 (14-45) | 26 (12-43) |
| Pain (VAS 0 -100 mm), med (IQR) | 40 (20-60) | 42 (20-60) |

*Legend:* Baseline characteristics of excluded patients due to study participation (n=337), compared to the study population. *p<0.05. Patients excluded due to study participation were slightly younger, more often ACPA-positive and had a higher disease activity at baseline.

*ACPA: anti-citrullinated protein antibody, DAS: Disease activity scores, ESR: estimated sedimentation rate, IQR: Interquartile range, med: median, n:number, SJC: swollen joint count, TJC: tender joint count, VAS: visual analogue scale.*

**Supplementary table S4: Results linear mixed model analysis of DAS over time**

| ***Table A.*** | **non-SDFR-development**  (n=632) | | **SDFR-development**  (n=149) | |
| --- | --- | --- | --- | --- |
| *Baseline DAS* | 3.18 | (3.10, 3.26) | 3.22 | (2.95, 3.48) |
| *Change in DAS (95%CI)* | | | | |
| ***0-4 months*** | **-0.96*** | **(-1.07, -0.85)** | **-1.59*** | **(-1.95, -1.24)** |
| *4-12 months* | -0.27 | (-0.37, -0.16) | -0.04 | (-0.40, +0.33) |
| *1-2 years* | -0.19 | (-0.29, -0.08) | -0.20 | (-0.58, +017) |
| *2-3 years* | +0.01 | (-0.12, +0.14) | -0.23 | (-0.70, +0.24) |
| *3-4 years* | +0.01 | (-0.13, +0.14) | 0.10 | (-0.43, +0.63) |
| *4-5 years* | -0.07 | (-0.21, +0.08) | -0.14 | (-0.82, +0.54) |
| *5-6 years* | -0.01 | (-0.17, +0.14) | -0.09 | (-0.60,+1.14) |
| *6-7 years* | +0.03 | (-0.13, +0.19) | +0.02 | (-2.29,+2.32) |

| ***Table B.*** | **ACPA-positive patients** | | | | **ACPA-negative patients** | | | |
| --- | --- | --- | --- | --- | --- | --- | --- | --- |
|  | No SDFR-development  (n=333) | | SDFR-development  (n=15) | | No SDFR-development  (n=273) | | SDFR-development  (n=127) | |
| *Baseline DAS* | 3.08 | (2.97, 3.19) | 2.97 | (2.35, 3.59) | 3.33 | (3.21, 3.45) | 3.28 | (2.94, 3.62) |
| *Change in DAS (95%CI)* | | | | | | | | |
| 0-4 months | -0.88 | (-1.03, -0.73) | -0.96 | (-1.77,-0.16) | **-1.07*** | **(-1.23, -0.90)** | **-1.73*** | **(-2.18, -1.28)** |
| 4-12 months | -0.23 | (-0.38, -0.08) | -0.02 | (-0.97, +0.93) | **-0.33*** | **(-0.49, -0.17)** | **+0.00*** | **(-0.45, +0.44)** |
| 1-2 years | -0.17 | (-0.32, -0.02) | -0.48 | (-1.47, +0.51) | -0.21 | (-0.36, -0.05) | -0.21 | (-0.66, +0.23) |
| 2-3 years | -0.05 | (-0.23, +0.23) | -0.27 | (-1.35, +0.81) | +0.06 | (-0.13, +0.25) | -0.20 | (-0.76, +0.36) |
| 3-4 years | -0.02 | (-0.20, +0.16) | -0.12 | (-1.41, +1.17) | +0.09 | (-0.11, +0.30) | +0.15 | (-0.49, +0.79) |
| 4-5 years | -0.09 | (-0.27, +0.09) | 0.22 | (-1.63, + 2.06) | -0.05 | (-0.28, +0.18) | -0.18 | (-0.98, +0.61) |
| 5-6 years | 0.01 | (-0.27, +0.28) |  |  | +0.00 | (-0.24, +0.24) | -0.13 | (-0.54, +1.44) |
| 6-7 years | 0.07 | (-0.15, +0.29) |  |  | -0.03 | (-0.28, +0.22) | -0.37 | (-2.69, +1.94) |

*Legend:*

**Table A)** Results of the LMM analysis of course of DAS over time for the total study population: i.e. those achieving SDFR (n=149) and those not (n=632). Patients achieving SDFR showed a statistically significant stronger DAS-decline within the first four months.

**Table B)** Results of the LMM analysis of course of DAS over time, stratified for ACPA-status. In ACPA-negative patient, patients achieving SDFR showed a statistically significant stronger DAS-decline within the first four months. This was effect was not present in ACPA-positive patients. Nevertheless, number of patients SDFR in ACPA-positive patients were small.

* Reflects statistical significant difference in course of DAS within a specific time interval between the non-SDFR-group and SDFR-group, e.g. a significant stronger decline between baseline in 4-months between both groups.

*ACPA: anti-citrullinated protein antibody, CI: confidence interval, DAS: Disease activity scores, SDFR: Sustained DMARD-free remission*

**Supplementary table S5: Results linear mixed model analysis of DAS-components over time**

**S5.1. Swollen joint count**

|  | **non-SDFR-development** | | **SDFR-development** | |
| --- | --- | --- | --- | --- |
| *Baseline SJC* | **7.40*** | **(6.92, 7.88)** | **9.18*** | **(7.62, 10.73)** |
| *Change in SJC (95%CI)* | | | | |
| ***0-4 months*** | **-4.31*** | **(-4.79, -3.82)** | **-7.46*** | **(-9.06, -5.87)** |
| *4-12 months* | -0.95 | (-1.40, -0.51) | -0.46 | (-1.96, +1.03) |
| *1-2 years* | -0.54 | (-0.99, -0.09) | -0.32 | (-1.90, +1.25) |
| *2-3 years* | +0.05 | (-0.49, +0.58) | -0.63 | (-2.61, +1.35) |
| *3-4 years* | -0.19 | (-0.72, +0.35) | +0.19 | (-1.95, +2.33) |
| *4-5 years* | -0.11 | (-0.68, +0.47) | +0.23 | (-2.42, +2.87) |
| *5-6 years* | +0.10 | (-0.53, +0.72) | +0.42 | (-3.72, +3.47) |
| *6-7 years* | +0.18 | (-0.46, +0.83) | +3.20 | (-6.54, +12.94) |

*Legend:* Results of the LMM analysis of course of swollen joint count over time for patients who achieved SDFR within 7 years compared to those who did not, in the total study population. Swollen joint count was slightly higher at baseline in patients who achieved SDFR within 7 years of follow-op. In the first 4 months, decline in the SDFR-group is significantly stronger compared to the non-SDFR-group.

* indicates a statistically significant difference in course of change in SJC.

*CI: confidence interval, SJC: swollen joint count, SDFR: sustained DMARD-free remission*

**S5.2. Tender joint count**

|  | **non-SDFR-development** | | **SDFR-development** | |
| --- | --- | --- | --- | --- |
| *Baseline TJC* | 7.50 | (7.03, 7.97) | 6.99 | (5.47, 8.50) |
| *Change in TJC (95%CI)* | | | | |
| ***0-4 months*** | **-3.33*** | **(-3.79, -2.87)** | **-4.79*** | **(-6.29, -3.29)** |
| *4-12 months* | -0.58 | (-1.01, -0.16) | +0.05 | (-1.36, +1.46) |
| *1-2 years* | -0.84 | (-1.27, -0.41) | -0.72 | (-2.23, +0.78) |
| *2-3 years* | -0.05 | (-0.56, +0.47) | -0.96 | (-2.87, +0.94) |
| *3-4 years* | -0.15 | (-0.66, +0.37) | +0.39 | (-1.68, +2.45) |
| *4-5 years* | -0.39 | (-0.95, +0.17) | +0.02 | (-2.55, +2.58) |
| *5-6 years* | +0.27 | (-0.35, +0.88) | +0.67 | (-2.18, +4.58) |
| *6-7 years* | -0.09 | (-0.72, +0.54) | -2.38 | (-11.92, +7.16) |

*Legend:* Results of the LMM analysis of course of tender joint count over time for patients who achieved SDFR within 7 years compared to those who did not, in the total study population. Decline in tender joint count was significantly stronger within the first 4 months in patients who achieved SDFR.

* indicates a statistically significant difference in course of change in TJC.

*CI: confidence interval, TJC: tender joint count, SDFR: sustained DMARD-free remission*

**S5.3. Estimated Sedimentation Rate (ESR)**

|  | **non-SDFR-development** | | **SDFR-development** | |
| --- | --- | --- | --- | --- |
| *Baseline ESR* | 34.13 | (32.07, 36.19) | 33.39 | (26.65, 40.14) |
| *Change in ESR (95%CI)* | | | | |
| ***0-4 months*** | **-12.54*** | **(-14.28, -10.80)** | **-19.32*** | **(-25.09, -13.54)** |
| *4-12 months* | -3.15 | (-4.78, -1.52) | -0.37 | (-5.83, +5.09) |
| *1-2 years* | -1.48 | (-3.10, +0.14) | +0.84 | (-4.76, +6.43) |
| *2-3 years* | +0.28 | (-1.70, +2.26) | -1.94 | (-9.15, +5.27) |
| *3-4 years* | +1.32 | (-0.73, +3.38) | -0.72 | (-8.96, +7.51) |
| *4-5 years* | -0.37 | (-2.65, +1.90) | -0.63 | (-10.84, +9.57) |
| *5-6 years* | +1.07 | (-1.40, +3.53) | +0.81 | (-15.40, +11.12) |
| *6-7 years* | +0.03 | (-2.53, +2.58) | +5.87 | (-28.72, +40.46) |

*Legend:* Results of the LMM analysis of course of ESR over time for patients who achieved SDFR within 7 years compared to those who did not, in the total study population. Decline in ESR was significantly stronger within the first 4 months in patients who achieved SDFR.

* indicates a statistically significant difference in course of change in ESR.

*CI: confidence interval, ESR: estimated sedimentation rate, SDFR: sustained DMARD-free remission*

**S5.4. Visual Analogue Scale (VAS)**

|  | **non-SDFR-development** | | **SDFR-development** | |
| --- | --- | --- | --- | --- |
| *Baseline VAS* | 41.81 | (36.94, 43.99) | 41.24 | (34.19, 48.30) |
| *Change in VAS (95%CI)* | | | | |
| *0-4 months* | -8.83 | (-11.58, -6.08) | -14.64 | (-23.64, -5.64) |
| *4-12 months* | -0.43 | (-3.16, +2.30) | +3.02 | (-6.02, +12.07) |
| *1-2 years* | -0.99 | (-3.77, +1.80) | -4.21 | (-13.79, +5.37) |
| *2-3 years* | +1.22 | (-2.13, +4.56) | +1.27 | (-10.99, +13.53) |
| *3-4 years* | -1.43 | (-4.78, +1.93) | -2.43 | (-15.91, +11.05) |
| *4-5 years* | +0.55 | (-3.09, +4.19) | +6.76 | (-9.69, +23.20) |
| *5-6 years* | +0.38 | (-3.60, +4.37) | +6.59 | (-22.04, +22.01) |
| *6-7 years* | +0.76 | (-3.34, +4.86) | -12.41 | (-70.39, +45.58) |

*Legend:* Results of the LMM analysis of course of VAS over time for patients who achieved SDFR within 7 years compared to those who did not, in the total study population. Nor baseline, nor change over time was significantly different between both groups. * indicates a statistically significant difference in course of change in VAS.

*CI: confidence interval, VAS: visual analogue scale, SDFR: sustained DMARD-free remission*

**Supplementary table S6: Logistic regression models (uni- and multivariable) for SDFR-development within 7 years**

|  | Odds ratio (95% CI) | | p-value |
| --- | --- | --- | --- |
| **Total study population** (n=772) |  | | |
| Age (years) | 1.04 (1.02 – 1.05) | 0.000 | |
| Gender (male) | 1.67 (1.15-2.41) | 0.007 | |
| Symptom duration <12w vs >12w (n=722) | 1.01 (0.69-1.48) | 0.974 | |
| ACPA status (n=748) | 0.10 (0.06-0.17) | 0.000 | |
| Baseline DAS (n=648) | 1.08 (0.88-1.32) | 0.458 | |
| ΔDAS_0-4m_ (n=365) | 0.56 (0.44-0.73) | 0.000 | |
| DAS_4m_ (n=365) | 0.54 (0.40-0.72) | 0.000 | |
| **ACPA** **negative patients** (n=400) |  | | |
| Age (years) | 1.02 (1.01-1.04) | 0.003 | |
| Gender (male) | 1.82 (1.17-2.82) | 0.008 | |
| Symptom duration <12w vs >12w (n=375) | 0.81 (0.52-1.26) | 0.353 | |
| Baseline DAS (n=340) | 0.98 (0.77-1.24) | 0.868 | |
| ΔDAS_0-4m_ (n=171) | 0.55 (0.39-0.75) | 0.000 | |
| DAS_4m_ (n=184) | 0.48 (0.33-0.68) | 0.000 | |
| **ACPA positive patients** (n=348) |  | | |
| Age | 1.05 (1.01-1.09) | 0.026 | |
| Gender | 0.87 (0.27-2.81) | 0.818 | |
| Symptom duration <12w vs >12w (n=325) | 0.40 (0.09-1.84) | 0.240 | |
| Baseline DAS (n=290) | 0.93 (0.51-1.70) | 0.819 | |
| ΔDAS_0-4m_ (n=163) | 0.97 (0.50-1.96) | 0.965 | |
| DAS_4m_ (n=169) | 0.92 (0.46-1.84) | 0.813 | |

**Table A. Univariable logistic regression models**

| **Table B. Multivariable logistic regression models** | | | | | | | | |
| --- | --- | --- | --- | --- | --- | --- | --- | --- |
|  | Odds ratio (95% CI) | | Odds ratio (95% CI) | | Odds ratio (95% CI) | | Odds ratio (95% CI) | |
| **Total study population** |  | |  | |  | |  | |
| Age | 1.02 (1.00-1.05) | 0.065 | 1.02 (1.00-1.05) | 0.065 | 1.02 (1.00-1.05) | 0.085 | 1.03 (1.00-1.05) | 0.023 |
| Gender (male) | 1.31 (0.67-2.56) | 0.428 | 1.31 (0.67-2.56) | 0.428 | 1.50 (0.78-2.86) | 0.221 | 1.20 (0.62-2.29) | 0.589 |
| ACPA positivity | 0.16 (0.07-0.35) | 0.000 | 0.16 (0.07-0.35) | 0.000 | 0.17 (0.07-0.38) | 0.000 | 0.13 (0.06-0.29) | 0.000 |
| Symptom duration (<12w) | 0.80 (0.41-1.53) | 0.489 | 0.80 (0.41-1.53) | 0.489 | 0.83 (0.44-1.58) | 0.578 | 0.89 (0.48-1.67) | 0.723 |
| Baseline DAS | 0.66 (0.45-0.99) | 0.042 | 1.38 (0.99-1.92) | 0.059 | - |  | - |  |
| DAS_4m_ | - |  | **0.48 (0.33-0.71)** | **0.000** | **-** |  | **0.54 (0.39-0.77)** | **0.001** |
| ΔDAS_0-4m_ | 0.48 (0.33-0.71) | 0.000 |  |  | **0.63 (0.48-0.82)** | **0.001** | - |  |
| **ACPA negative patients** |  | |  | |  | |  | |
| Age | 1.02 (0.99-1.04) | 0.380 | 1.02 (0.99-1.04) | 0.380 | 1.01 (0.99-1.04) | 0.369 | 1.02 (0.99-1.05) | 0.129 |
| Gender (male) | 1.40 (0.66-2.97) | 0.308 | 1.40 (0.66-2.97) | 0.308 | 1.65 (0.80-3.39) | 0.178 | 1.25 (0.61-2.57) | 0.545 |
| Symptom duration (<12w) | 0.87 (0.42-1.79) | 0.698 | 0.87 (0.42-1.79) | 0.698 | 0.93 (0.45-1.88) | 0.822 | 0.97 (0.49-1.93) | 0.924 |
| Baseline DAS | 0.63 (0.42-0.99) | 0.043 | 1.44 (1.00-2.08) | 0.048 |  |  |  |  |
| DAS_4m_ | **-** |  | **0.44 (0.29-0.68)** | **0.000** | **-** |  | **0.51 (0.35-0.75)** | **0.000** |
| ΔDAS_0-4m_ | **0.44 (0.29-0.68)** | **0.000** | **-** |  | **0.58 (0.42-0.80)** | **0.001** | - |  |
| **ACPA positive patients** |  | |  | |  | |  | |
| Age | 1.06 (1.01-1.13) | 0.038 | 1.06 (1.01-1.13) | 0.038 | 1.06 (1.00-1.13) | 0.042 | 1.07 (1.00-1.13) | 0.037 |
| Gender (male) | 1.10 (0.23-5.18) | 0.907 | 1.10 (0.23-5.18) | 0.907 | 1.16 (0.25-5.32) | 0.848 | 1.10 (0.23-5.18) | 0.903 |
| Symptom duration (<12w) | 0.52 (0.10-2.84) | 0.449 | 0.52 (0.10-2.84) | 0.449 | 0.51 (0.09-2.79) | 0.438 | 0.54 (0.10-2.87) | 0.469 |
| Baseline DAS | 0.78 (0.30-2.10) | 0.620 | 1.00 (0.40-2.50) | 0.997 | - |  | - |  |
| DAS_4m_ | **-** |  | **0.78 (0.32-1.91)** | **0.587** | - |  | **0.80 (0.36-1.80)** | **0.592** |
| ΔDAS_0-4m_ | **0.78 (0.32-1.91)** | **0.587** | **-** |  | **1.04 (0.52-2.10)** | **0.909** | **-** |  |

*Legend:* Logistic regression models, univariable (table A) and multivariable (table B) with SDFR (yes/no) as dependent outcome variable. Analysis were presented for total study population, and stratified for ACPA status. DAS: Disease activity score, BL: baseline, CI: Confidence interval.

**Supplementary table S7: Sensitivity analysis of all patients achieving SDFR during complete follow-up**

|  | **Total study population** | | | **ACPA-positive patients** | | **ACPA-negative patients** | |
| --- | --- | --- | --- | --- | --- | --- | --- |
|  | No SDFR  (n=599) | | SDFR  (n=173) | No SDFR  (n=330) | SDFR  (n=18) | No SDFR  (n=252) | SDFR  (n=148) |
| *Baseline DAS* | 3.19  (3.11-3.28) | | 3.17  (2.92-3.43) | 3.10  (2.98, 3.21) | 2.84  (2.25, 3.42) | 3.36  (3.24, 3.49) | 3.24  (2.91, 3.58) |
| *Change DAS over time* | |  |  |  |  |  |  |
| 0-4 months | **-0.98***  **(-1.07, -0.85)** | | **-1.46***  **(-1.95, -1.24)** | -0.90  (-1.05, -0.75) | -0.92  (-1.70, -0.14) | **-1.13***  **(-1.29, -0.96)** | **-1.58***  **(-2.02, -1.14)** |
| 4-12 months | **-0.27***  **(-0.37, -0.16)** | | **-0.02***  **(-0.40, +0.33)** | -0.23  (-0.39, -0.08) | +0.14  (-0.75, +1.02) | **-033***  **(-0.50, -0.17)** | **-0.01***  **(-0.46, +0.43)** |
| 1-2 years | -0.17  (-0.29, -0.08) | | -0.30  (-0.58, +0.17) | -0.15  (-0.31, +0.00) | -0.75  (-1.66, +0.15) | -0.17  (-0.33, -0.01) | -0.27  (-0.71, +0.17) |
| 2-3 years | +0.01  (-0.11, +0.15) | | -0.20  (-0.70, +0.24) | -0.04  (-0.22, +0.14) | -0.09  (-1.13, +0.95) | +0.09  (-0.11, +0.28) | -0.20  (-0.76, +0.36) |
| 3-4 years | +0.02  (-0.13, +0.14) | | +0.25  (-0.43, +0.63) | -0.03  (-0.21, +0.15) | -0.14  (-1.43, +1.15) | +0.04  (-0.18, +0.26) | +0.28  (-0.34, +0.89) |
| 4-5 years | -0.05  (-0.21, +0.08) | | -0.18  (-0.82, +0.54) | -0.07  (-0.27, +0.13) | +0.00  (-1.50, +1.50) | -0.04  (-0.29, +0.21) | -0.19  (-0.90, +0.53) |
| 5-6 years | -0.01  (-0.17, +0.19) | | -0.14  (-0.60, +1.14) | -0.06  (-0.28, +0.16) | +0.01  (-1.10, +1.86) | +0.01  (-0.26, +0.28) | -0.14  (-0.72, +0.86) |
| 6-7 years | +0.04  (-0.13, +0.19) | | +0.02  (-2.29, +2.32) | +0.16  (-0.03, +0.35) | -0.30  (-1.66, +1.06) | -0.01  (-0.25, +0.24) | +0.01  (-0.75, +0.78) |

*Legend:* LMM analysis of changes in disease activity over 7 years of follow-up compared between patients achieving SDFR and those not achieving SDFR. SDFR was defined as achieving SDFR during entire follow-up, instead of restriction to 7 year like in primary analysis. Absolute DAS at baseline (95%CI), and change within each time-interval (95%CI) are shown. * indicates a significant different course of DAS within the specific interval between both groups.

*ACPA: anti-citrullinated protein antibody, DAS: Disease activity score, SDFR: Sustained DMARD-free remission*

**Supplementary table S8: Sensitivity analysis of patients achieving SDFR within 7-years, but flaring after 7-years.**

|  | **Total study population** | | | **ACPA-positive patients** | | **ACPA-negative patients** | |
| --- | --- | --- | --- | --- | --- | --- | --- |
|  | No SDFR  (n=599) | | SDFR  (n=173) | No SDFR  (n=330) | SDFR  (n=18) | No SDFR  (n=252) | SDFR  (n=148) |
| *Baseline DAS* | 3.20  (3.12-3.29) | | 3.18  (2.92-3.44) | 3.09  (2.98, 3.20) | 2.84  (2.26, 3.41) | 3.35  (3.22, 3.48) | 3.25  (2.92, 3.59) |
| *Change DAS over time* | |  |  |  |  |  |  |
| 0-4 months | **-0.99***  **(-1.10, -0.88)** | | **-1.47***  **(-1.82, -1.13)** | -0.88  (-1.03, -0.74) | -0.94  (-1.72, -0.15) | **-1.11***  **(-1.28, -0.96)** | **-1.58***  **(-2.03, -1.13)** |
| 4-12 months | -0.27  (-0.38, -0.16) | | -0.02  (-0.38, +0.33) | -0.24  (-0.39, -0.09) | +0.14  (-0.74, +1.02) | **-0.32***  **(-0.49, -0.16)** | **-0.02***  **(-0.46, +0.42)** |
| 1-2 years | -0.16  (-0.27, -0.05) | | -0.30  (-0.66, +0.07) | -0.15  (-0.30, +0.00) | -0.77  (-1.68, +0.13) | -0.18  (-0.34, -0.02) | -0.28  (-0.72, +0.16) |
| 2-3 years | +0.01  (-0.12, +0.15) | | -0.20  (-0.65, +0.26) | -0.06  (-0.24, +0.12) | -0.05  (-1.08, +0.99) | +0.09  (-0.11, +0.28) | -0.20  (-0.75, +0.36) |
| 3-4 years | -0.02  (-0.15, +0.12) | | +0.25  (-0.26, +0.72) | -0.03  (-0.20, +0.15) | -0.14  (-1.42, +1.13) | +0.05  (-0.17, +0.27) | +0.28  (-0.33, +0.88) |
| 4-5 years | -0.07  (-0.22, +0.09) | | -0.18  (-0.75, +0.40) | -0.06  (-0.26, +0.13) | +0.00  (-1.48, +1.48) | -0.02  (-0.27, +0.23) | -0.16  (-0.87, +0.55) |
| 5-6 years | -0.04  (-0.20, +0.13) | | -0.14  (-0.58, +0.68) | -0.04  (-0.25, +0.18) | +0.03  (-1.15, +1.78) | +0.03  (-0.24, +0.30) | -0.11  (-0.65, +0.91) |
| 6-7 years | +0.09  (-0.06, +0.24) | | +0.02  (-0.61, +0.62) | +0.09  (-0.13, +0.31) | -0.19  (-1.66, +1.27) | -0.04  (-0.32, +0.24) | +0.01  (-0.86, +0.61) |

*Legend:* LMM analysis of changes in disease activity over 7 years of follow-up compared between patients achieving SDFR within 7 years (including patients with a flare after 7 years) and those not achieving SDFR. In this, patients achieving SDFR within 7 years, but with a flare after 7 years were included in the SDFR-group. Absolute DAS at baseline (95%CI), and change within each time-interval (95%CI) are shown. * indicates a significant different course of DAS within the specific interval between both groups.

*ACPA: anti-citrullinated protein antibody, DAS: Disease activity score, SDFR: Sustained DMARD-free remission*

**Supplementary table S9: Logistic regression models (uni- and multivariable) SDFR within 7 years using imputed data**

| **Table A. Univariable logistic regression** | | | | |
| --- | --- | --- | --- | --- |
|  |  | Odds ratio (95% CI) | p-value | |
| **Total study population** (n=772) | |  | | |
| Age | | 1.04 (1.02-1.05) | | 0.000 |
| Gender | | 1.67 (1.15-3.41) | | 0.007 |
| Symptom duration at diagnosis  *(≤12w vs >12w)* | | 1.02 (0.79-1.50) | | 0.925 |
| ACPA status | | 0.10 (0.06-0.17) | | 0.000 |
| Baseline DAS | | 1.06 (0.88-1.27) | | 0.562 |
| DAS_4months_ | | 0.59 (0.46-0.75) | | 0.000 |
| ΔDAS_0-4m_ | | 0.65 (0.54-0.79) | | 0.000 |
| **ACPA negative patients** (n=400) | |  | | |
| Age | | 1.02 (1.01-1.04) | | 0.003 |
| Gender (male) | | 1.82 (1.17-2.82) | | 0.008 |
| Symptom duration at diagnosis  *(≤12w vs >12w)* | | 0.83 (0.53-1.29) | | 0.406 |
| Baseline DAS | | 0.97 (0.78-1.21) | | 0.775 |
| DAS_4months_ | | 0.58 (0.43-0.76) | | 0.000 |
| ΔDAS_0-4m_ | | 0.69 (0.56-0.85) | | 0.001 |
| **ACPA positive patients** (n=348) | |  | | |
| Age | | 1.05 (1.01-1.09) | | 0.026 |
| Gender | | 0.87 (0.27-2.81) | | 0.818 |
| Symptom duration at diagnosis  *(≤12w vs >12w)* | | 0.42 (0.09-1.92) | | 0.265 |
| Baseline DAS | | 0.82 (0.45-1.48) | | 0.504 |
| DAS_4months_ | | 0.74 (0.40-1.40) | | 0.359 |
| ΔDAS_0-4m_ | | 0.91 (0.52-1.57) | | 0.725 |

| **Table B. Multivariable logistic regression models** | | | | | | | | |
| --- | --- | --- | --- | --- | --- | --- | --- | --- |
|  | Odds ratio (95% CI) |  | Odds ratio (95% CI) |  | Odds ratio (95%CI) |  | Odds ratio (95% CI) |  |
| **Total study population** | | | | | | | | |
| Age | 1.03 (1.01-1.04) | 0.001 | 1.03 (1.01-1.04) | 0.001 | 1.02 (1.01-1.04) | 0.001 | 1.03 (1.01-1.04) | 0.000 |
| Gender (male) | 1.20 (0.78-1.87) | 0.427 | 1.20 (0.78-1.87) | 0.427 | 1.33 (0.87-2.05) | 0.187 | 1.20 (0.77-1.87) | 0.411 |
| Symptom duration at diagnosis *(≤12w vs >12w)* | 0.61 (0.39-0.96) | 0.033 | 0.61 (0.39-0.96) | 0.033 | 0.62 (0.39-0.96) | 0.033 | 0.63 (0.40-0.99) | 0.044 |
| ACPA positivity | 0.11 (0.06-0.19) | 0.000 | 0.11 (0.06-0.19) | 0.000 | 0.11 (0.06-0.20) | 0.000 | 0.11 (0.06-0.19) | 0.000 |
| Baseline DAS | 0.64 (0.48-0.86) | 0.003 | 1.14 (0.90-1.44) | 0.277 | - |  | - |  |
| DAS_4months_ | - |  | 0.56 (0.42-0.75) | 0.000 | - |  | 0.59 (0.45-0.77) | 0.000 |
| ΔDAS_0-4m_ | 0.56 (0.42-0.75) | 0.000 | - |  | 0.72 (0.59-0.88) | 0.001 | - |  |
| **ACPA negative patients** | | | | | | | | |
| Age | 1.02 (1.01-1.04) | 0.007 | 1.02 (1.01-1.04) | 0.007 | 1.02 (1.00-1.04) | 0.005 | 1.02 (1.00-1.04) | 0.005 |
| Gender (male) | 1.33 (0.82-2.15) | 0.247 | 1.33 (0.82-2.15) | 0.247 | 1.34 (0.83-2.16) | 0.230 | 1.34 (0.83-2.16) | 0.230 |
| Symptom duration at diagnosis *(≤12w vs >12w)* | 0.64 (0.40-1.04) | 0.074 | 0.64 (0.40-1.04) | 0.074 | 0.68 (0.42-1.09) | 0.104 | 0.68 (0.42-1.09) | 0.104 |
| Baseline DAS | 0.64 (0.47-0.89) | 0.007 | 1.18 (0.92-1.52) | 0.007 | - |  | - |  |
| DAS_4months_ | - | - | 0.55 (0.40-0.75) | 0.000 | - |  | 0.58 (0.43-0.78) | 0.000 |
| ΔDAS_0-4m_ | 0.55 (0.40-0.75) | 0.000 | - | - | 0.70 (0.56-0.87) | 0.001 |  |  |
| **ACPA positive patients** | | | | | | | | |
| Age | 1.08 (1.02-1.14) | 0.005 | 1.08 (1.02-1.14) | 0.005 | 1.05 (1.01-1.10) | 0.017 | 1.06 (1.01-1.10) | 0.013 |
| Gender (male) | 0.63 (0.19-2.15) | 0.464 | 0.63 (0.19-2.15) | 0.464 | 0.71 (0.22-2.35) | 0.576 | 0.64 (0.19-2.17) | 0.474 |
| Symptom duration at diagnosis *(≤12w vs >12w)* | 0.35 (0.08-1.65) | 0.185 | 0.35 (0.08-1.65) | 0.185 | 0.35 (0.08-1.64) | 0.184 | 0.35 (0.08-1.63) | 0.181 |
| Baseline DAS | 0.60 (0.28-1.32) | 0.207 | 0.84 (0.42-1.67) | 0.612 | - |  | - |  |
| DAS_4months_ | - |  | 0.72 (0.35-1.51) | 0.386 | - |  | 0.68 (0.35-1.34) | 0.264 |
| ΔDAS_0-4m_ | 0.72 (0.35-1.51) | 0.386 | - |  | 0.92 (0.53-1.60) | 0.757 |  |  |

*Legend:* Logistic regression models, univariable (table A) and multivariable (table B), with SDFR (yes/no) as dependent outcome variable.

*ACPA: anti-citrullinated protein antibody, DAS: Disease activity score, BL: baseline, CI: Confidence interval*

**Supplementary figure S10: Kaplan Meier analysis using imputed data**


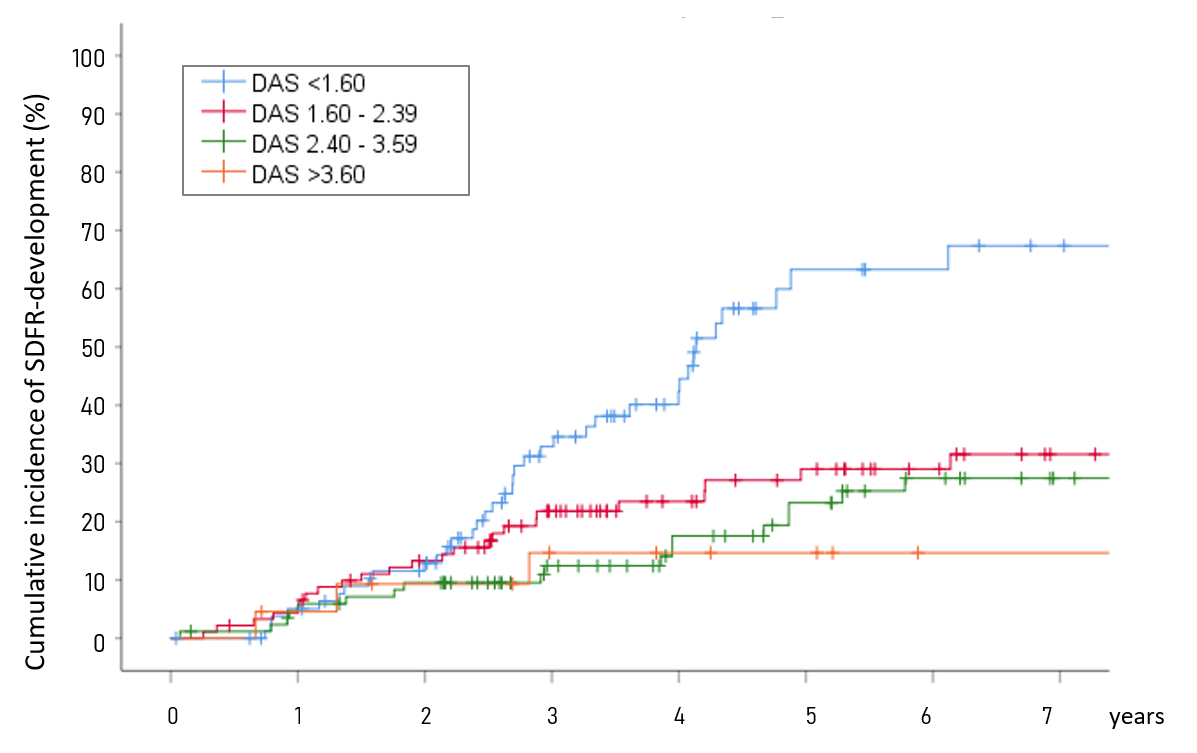


*Legend:* Cumulative survival proportion within 7 years per DAS-category. Cumulative survival proportions were log-transformed since these were non-normally distributed and subsequently averaged. Cumulative incidence was calculated by 1-survival proportion. One cumulative incidence curve was randomly chosen from all 30 imputations for presentation in these supplementary materials.

*DAS: Disease activity score, SDFR: Sustained DMARD-free remission*
